# Inter domain linker region affects properties of CBM6 in GH5_34 arabinoxylanases and alters oligosaccharide product profile

**DOI:** 10.1093/glycob/cwae048

**Published:** 2024-07-10

**Authors:** Siri Norlander, Andrius Jasilionis, Leila Allahgholi, Christina Wennerberg, Carl Grey, Patrick Adlercreutz, Eva Nordberg Karlsson

**Affiliations:** Division of Biotechnology, Department of Chemistry, Lund University, PO Box 124, Lund SE-221 00, Sweden; Division of Biotechnology, Department of Chemistry, Lund University, PO Box 124, Lund SE-221 00, Sweden; Division of Biotechnology, Department of Chemistry, Lund University, PO Box 124, Lund SE-221 00, Sweden; Division of Biotechnology, Department of Chemistry, Lund University, PO Box 124, Lund SE-221 00, Sweden; Division of Biotechnology, Department of Chemistry, Lund University, PO Box 124, Lund SE-221 00, Sweden; Division of Biotechnology, Department of Chemistry, Lund University, PO Box 124, Lund SE-221 00, Sweden; Division of Biotechnology, Department of Chemistry, Lund University, PO Box 124, Lund SE-221 00, Sweden

**Keywords:** affinity gel electrophoresis, arabinoxylanase, carbohydrate binding module, domain swapping

## Abstract

Understanding the relation between enzyme domain structure and catalytic activity is crucial for optimal engineering of novel enzymes for lignocellulose bioconversion. Xylanases with varying specificities are commonly used to valorise the hemicellulose arabinoxylan (AX), yet characterization of specific arabinoxylanases remain limited. Two homologous GH5_34 arabinoxylanases, *Hh*Xyn5A and *Ct*Xyn5A, in which the two domains are connected by a 40-residue linker, exhibit distinct activity on AX, yielding different reaction product patterns, despite high sequence identity, conserved active sites and similar domain composition. In this study, the carbohydrate binding module 6 (CBM6), or the inter domain linker together with CBM6, were swapped to investigate their influence on hydrolytic activity and oligosaccharide product pattern on cereal AXs. The variants, with only CBM6 swapped, displayed reduced activity on commercial wheat and rye AX, as well as on extracted oat fibre, compared to the original enzymes. Additionally, exchange of both linker and CBM6 resulted in a reduced ratio of enzyme produced in soluble form in *Escherichia coli* cultivations, causing loss of activity of both *Hh*Xyn5A and *Ct*Xyn5A variants. Analysis of oligosaccharide product patterns applying HPAEC–PAD revealed a decreased number of reaction products for *Ct*Xyn5A with swapped CBM6, which resembled the product pattern of *Hh*Xyn5A. These findings emphasize the importance of the CBM6 interactions with the linker and the catalytic domain for enzyme activity and specificity, and underlines the role of the linker in enzyme structure organisation and product formation, where alterations in linker interactions with the catalytic and/or CBM6 domains, influence enzyme-substrate association and specificity.

## Introduction

Glycoside hydrolases (GH) contribute to plant biomass hydrolysis by degrading a diverse range of polysaccharides. In the Carbohydrate Active enZymes (CAZy) database (Drula et al. 2022), numerous GH families are listed. Among these families, GH5 is a large family, widely distributed across archaea, bacteria and eukaryota, hosting enzymes with a variety of specificities, including for example endo- and exo-glucanases, endo- and exomannanases, 1,6-galactanases, xyloglucanases and arabinoxylanases (Drula et al. 2022). This large variation is reflected in the sequences, and has resulted in a GH5 subfamily classification that delineates family members into a number of monospecific and polyspecific clades (Aspeborg et al. 2012). Enzymes specifically targeting xylans can be found in different GH5 subfamilies, where exclusive arabinoxylanases are found in the small GH5_34 subfamily (Correia et al. 2011; Labourel et al. 2016; Norlander et al. 2023). The arabinoxylanases attributed to the GH5_34 subfamily require an α-1,3-Ara*f* substitution in the −1 subsite position in their active site for the catalytic hydrolysis to occur (Correia et al. 2011; Labourel et al. 2016). Their relatively open catalytic cleft allows Ara*f* substitutions in all subsites from −2 to +2 including double substitutions, making them interesting for the production of arabinose substituted products (Correia et al. 2011; Falck et al. 2018; Norlander et al. 2023). Despite being of great interest for the production of arabinoxylo-oligosaccharides, only a few subfamily members have been studied in detail (Correia et al. 2011; Labourel et al. 2016). The most well studied GH5_34 arabinoxylanase *Ct*Xyn5A from *Acetivibrio thermocellus* (initially *Clostridium thermocellum*) is a multi-modular enzyme with three carbohydrate binding modules (CBM6, CBM13, and CBM62), which are suspected to aid in the hydrolysis of complex heteroxylans (Labourel et al. 2016). Attempts to produce the single catalytic module of *Ct*Xyn5A have been unsuccessful, while a truncated variant consisting of the catalytic GH5 module and a CBM6 has been successfully produced in *Escherichia coli* as well as structurally characterized (PDB 2Y8K) (Correia et al. 2011). The novel thermostable GH5_34 subfamily arabinoxylanase *Hh*Xyn5A from *Herbinix hemicellulosilytica* has recently been extensively characterized (Norlander et al. 2023). Analogously to *Ct*Xyn5A, *Hh*Xyn5A is also a multi-modular enzyme, where the two-domain enzyme variant consisting of catalytic domain and CBM6 is the smallest functional entity. Interestingly, in contrast to the commercially available two-domain *Ct*Xyn5A variant, the recently characterised *Hh*Xyn5A produces a different oligosaccharide product profile, which may be influenced by the atypical CBM6 that is significantly less conserved than the catalytic GH5 domain (Norlander et al. 2023).

CBM6 modules display the typical β-sandwich fold of the jelly roll superfamily (consisting of two sheets of antiparallel β-strands connected by loops) and is in general found in polysaccharide degrading enzymes from archaea or bacteria (Michel et al. 2009), but has, however, also been reported to be associated with enzymes originating from eukaryotes (Takaki et al. 2002). CBM6 can be found attached to enzymes with varying substrate specificities (e.g. xylanases, cellulases, agarases, laminarinases) (Michel et al. 2009), with reported binding specificities for linear and branched/decorated xylan, β-1,4-glucan (or cellulose), mixed-linked β-1,3-1,4-glucan (or lichenan), agarose, β-1,3-glucan (or laminarin) or chitin. The first characterized CBM6 originated from a multi-modular xylanase from *C. thermocellum* (Fernandes et al. 1999). Subsequent sequence analyses of other CBM6 structures indicated a large diversity in ligand specificity, and phylogenetic analyses of CBM6 sequences resulted in module family division into subfamilies (Czjzek et al. 2001; Abbott et al. 2009). The ligand binding site of CBM6 has either been located as a shallow cleft on the concave surface of the β-sheets (as observed in CBMs from different families), or more commonly, located within the connecting loops of the two β-sheets (the apex site or site A, in e.g. PDB 1GMM; PDB 1UXX; PDB 1NAE; PDB 1W9W). Moreover, two distinct binding sites were revealed in a CBM6 of lichenase from *Cellvibrio mixtus* (PDB 1UYY; PDB 1UY0; PDB 1UYZ) that displayed binding affinities for both sites (Henshaw et al. 2004; Pires et al. 2004). In *Ct*Xyn5A, the binding site of the CBM6 is located on the loops (binding site A) and is including a pair of aromatic residues, proposed as a central feature of this site (Correia et al. 2011). In contrast, the CBM6 of *Hh*Xyn5A is missing these residues, suggesting that binding is mediated by other residues and interactions, or occurring at another site of the module, as proposed by docking simulations (Norlander et al. 2023).

The described difference raised interest in the possibilities to combine the CBM6 and catalytic modules from the two GH5_34 enzymes, to investigate the influence of the exchange on the activity profiles. *Hh*Xyn5A and *Ct*Xyn5A displayed different oligosaccharide product profiles on arabinoxylan (AX) substrates, and this was suggested to be attributed to the structural differences between CBM6 domains of the enzymes (Norlander et al. 2023). In this study, the CBM6 between *Hh*Xyn5A and *Ct*Xyn5A were swapped (keeping the original interactions of the catalytic domain and the linker), as well as the linker region together with the CBM6 (keeping the interactions with the original linker and CBM6), to investigate and compare the influence of the CBM6 and the linker on catalytic activity and substrate affinity for these two homologous GH5_34 arabinoxylanases.

## Results

### Construction, production and purification of *Hh*Xyn5A and *Ct*Xyn5A enzyme variants

**Fig. 1:**
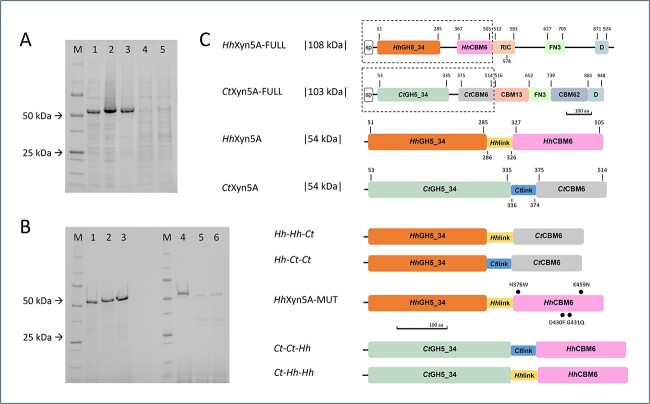
A) SDS–PAGE of recombinant enzyme variants in lysate before purification. (M) – Precision plus protein unstained standards (bio-rad) molecular-mass marker, (1) *Hh*Xyn5A, (2) *Hh-Hh-Ct*, (3) *Hh-Ct-Ct*, (4) *Ct-Ct-Hh*, (5) *Ct-Hh-Hh*. B) SDS–PAGE of recombinant enzyme variants after purification and dialysis. (M) – Precision plus protein unstained standards (bio-rad) molecular-mass marker, (1) *Hh*Xyn5A, (2) *Hh-Hh-Ct*, (3) *Hh-Ct-Ct*, (4) *Ct*Xyn5A, (5) *Ct-Ct-Hh*, (6) *Ct-Hh-Hh*. C) Schematic representation of the domain organization of *Hh*Xyn5A and *Ct*Xyn5A enzyme variants in comparison with reference full-length multi-modular *Hh*Xyn5A-FULL (Norlander et al. 2023) and *Ct*Xyn5A-FULL (Montanier et al. 2011; Brás et al. 2011; Norlander et al. 2023) organisations. sp = signal peptide; GH = glycoside hydrolase; CBM = carbohydrate binding module; RIC = ricin-type beta-trefoil lectin domain; FN3 = fibronectin type 3 like domain; D = dockerin domain. Dotted lines surrounding domains that are included in the truncated two-domain variants used in this study. Numbering of the domains refers to the amino acid sequence interval. Indicated mutations are highlighted as bold dots.

Four variants of the two-domain enzymes *Hh*Xyn5A and *Ct*Xyn5A, consisting of the catalytic GH5_34 domain, inter domain linker region and the CBM6, were constructed from the respective amino acid sequences, with swapped CBM6, or swapped linker region and CBM6 (Fig. 1C). The resulting variants were termed indicating (from N-terminus to C-terminus) catalytic module-linker region-CBM6 for simplicity naming modules and linker region *Hh* or *Ct* according to their respective origin. The linker region natively present between the two modules was clearly identified in both *Ct*Xyn5A and *Hh*Xyn5A via determination of the domain boundaries of the respective enzyme, using a combination of available sequence annotations and tertiary structure comparisons (as described in Materials and methods). The *Hh*Xyn5A inter domain linker region constituted a stretch of 41 amino acids (Thr286-Thr326), while the corresponding region in *Ct*Xyn5A was 39 amino acids (Gly336-Thr374) (Supplementary Fig. S1). A mutated variant of *Hh*Xyn5A was also constructed by changing four residues of the *Hh*CBM6 into the residue found at the corresponding position in *Ct*CBM6 (H376W, D430F, G431Q, K459N), in order to investigate the influence of the exchange on the enzyme activity profile. The four mutated amino acid residues were identified in cleft A of the *Ct*CBM6 as structurally essential for substrate binding and specificity of *Ct*Xyn5A (Correia et al. 2011; Norlander et al. 2023).

Production of the four domain-swapped enzyme variants (Fig. 1C), along with *Hh*Xyn5A (Norlander et al. 2023), resulted in high yields of variants *Hh-Hh-Ct* (*Hh*GH5_34-*Hh*Linker-*Ct*CBM6) and *Hh-Ct–Ct* (*Hh*GH5_34-*Ct*Linker-*Ct*CBM6) (0.12 and 0.16 g/L culture, respectively), whereas production yields of variants *Ct-Ct-Hh* (*Ct*GH5_34-*Ct*Linker-*Hh*CBM6) and *Ct-Hh-Hh* (*Ct*GH5_34-*Hh*Linker-*Hh*CBM6) (0.03 and 0.06 g/L culture, respectively) were comparatively low (Fig. 1A). A single-step purification applying immobilized metal ion affinity chromatography (IMAC) of soluble recombinant proteins resulted in a purity near homogeneity of all four variants (Fig. 1B). The pure *Hh-Hh-Ct* variant was stable in solution in both elution and formulation buffers, while the *Hh-Ct-Ct* variant aggregated already in the elution buffer. Domain splitting was observed for variants *Ct-Ct-Hh* as well as *Ct-Hh-Hh*, most probably caused by endogenous expression host proteases (Fig. 1B and Supplementary Fig. S2). Oligopeptides of the purified variants *Ct-Ct-Hh* and *Ct-Hh-Hh* fragments, identified applying mass spectrometry (MS) analysis, confirmed proteolytic cleavages in the linker region sequence as most likely (Supplementary Fig. S2). Production of the mutated *Hh*Xyn5A-MUT variant (*Hh*GH5_34-*Hh*Linker-*Hh*CBM6(H376W, D430F, G431Q, K459N)) (Fig. 1C) resulted in a high production level of recombinant protein purified to near homogeneity applying IMAC (data not shown). The pure *Hh*Xyn5A-MUT variant was stable in solution in both elution and formulation buffers. Observed differences in heterologous production of the enzyme variants in the soluble protein fraction and in protease susceptibility between the variants indirectly indicate a structural importance of the interactions between the catalytic domain and the linker region for protein solubility and integrity of the produced enzyme, as both *Hh*Xyn5A and *Hh-Hh-Ct* variants were demonstrated to be produced in the soluble protein fraction in the cultivations (Table 3).

**Table 3:** Enzyme variant characteristics.

**enzyme variant**	**production level (g/L)**	**protease susceptibility**	**solubility**	**thermostability (*T***_**m**_ **,°C)**	**enzymatic activity** ^a^	**HPAEC–PAD pattern**	**WAX affinity**	**linker region interactions**
*Hh*Xyn5A	high (0.15)^b^	not susceptible	soluble	thermostable (~60)	+++++	fewer peaks than *Ct*Xyn5A	low	NA
*Hh-Hh-Ct*	high (0.12)	not susceptible	soluble	> *Hh*Xyn5A	+++	no product peaks detected	> *Hh*Xyn5A	9 differences
*Hh-Ct-Ct*	high (0.16)	not susceptible	aggregation-prone	> *Hh*Xyn5A	++	no product peaks detected	> *Hh*Xyn5A	9 differences
*Hh*Xyn5A-MUT	high^c^ (ND)	not susceptible	soluble	≈ *Hh*Xyn5A	++++	similar peak pattern to *Hh*Xyn5A	ND	ND
*Ct*Xyn5A	high^d^ (ND)	not susceptible	aggregation-prone	thermostable (~75)	+++++	many undefined peaks detected	low	NA
*Ct-Ct-Hh*	low (0.03)	susceptible	soluble	> *Ct*Xyn5A	++++	similar peak pattern to *Hh*Xyn5A	< *Ct*Xyn5A	5 differences
*Ct-Hh-Hh*	low (0.06)	susceptible	soluble	> *Ct*Xyn5A	+	no product peaks detected	< *Ct*Xyn5A	6 differences

^a^Scale of enzymatic activity is based on DNS and HPAEC–PAD results

^b^(Norlander et al. 2023)

^c^Based on SDS–PAGE visualization

^d^Based on SDS–PAGE visualization (Correia et al. 2011); NA = not applicable; ND = not determined.

### Enzyme variant thermostability

The thermostability of the *Hh*Xyn5A and *Ct*Xyn5A variants were investigated by measuring melting temperature (*T*_m_) applying nano scale differential scanning fluorimetry (nanoDSF) at pH 6.5 (Table 1). The *Hh*Xyn5A was unfolding at approximately 60 °C, whereas *Ct*Xyn5A was more thermostable demonstrating a *T*_m_ of approximately 75 °C. The difference in thermostability between *Hh*Xyn5A and *Ct*Xyn5A, at the optimal pH 6.5, was in accordance with previous data, where a *T*_m_ of 61 and 73 °C, respectively, was obtained (Norlander et al. 2023). The *Hh*Xyn5A-MUT variant, with four amino acid residues mutated in the sequence of *Hh*CBM6, displayed a thermostability similar to *Hh*Xyn5A, with a *T*_m_ slightly lower than 62 °C. The domain-swapped variants *Hh*-*Hh*-*Ct* and *Hh*-*Ct*-*Ct* were more thermostable than *Hh*Xyn5A with a Δ*T*_m_ of 3.3–3.6 °C, respectively, indicating stabilisation of the variant structures by including *Ct*CBM6. Inclusion of the *Hh*CBM6 demonstrated a negligible increase in thermostability as the *T*_m_ of *Ct*-*Ct*-*Hh* and *Ct*-*Hh*-*Hh* increased only by 1.0–1.4 °C, respectively, as compared with the thermostability of the original *Ct*Xyn5A (Table 1).

**Table 1:** Melting temperatures (*T*_m_) ± standard deviation of the enzyme variants in reaction buffer (10 mM HEPES-HCl pH 6.5 (RT)) determined by the nano scale differential scanning fluorimetry. Measurements were performed in triplicates and *T*_m_ was extracted from the first derivative of the absorbance ratio 350/330 nm.

**enzyme variant**	**melting temperature (*T***_**m**_ **,°C)**
*Hh*Xyn5A	59.9 ± 0.0
*Hh-Hh-Ct*	63.5 ± 0.4
*Hh-Ct-Ct*	63.2 ± 0.0
*Hh*Xyn5A-MUT	61.8 ± 0.1
*Ct*Xyn5A	74.7 ± 0.2
*Ct-Ct-Hh*	76.1 ± 0.1
*Ct-Hh-Hh*	75.7 ± 0.5

### Enzyme variant activity and oligosaccharide product profiles

**Fig. 2:**
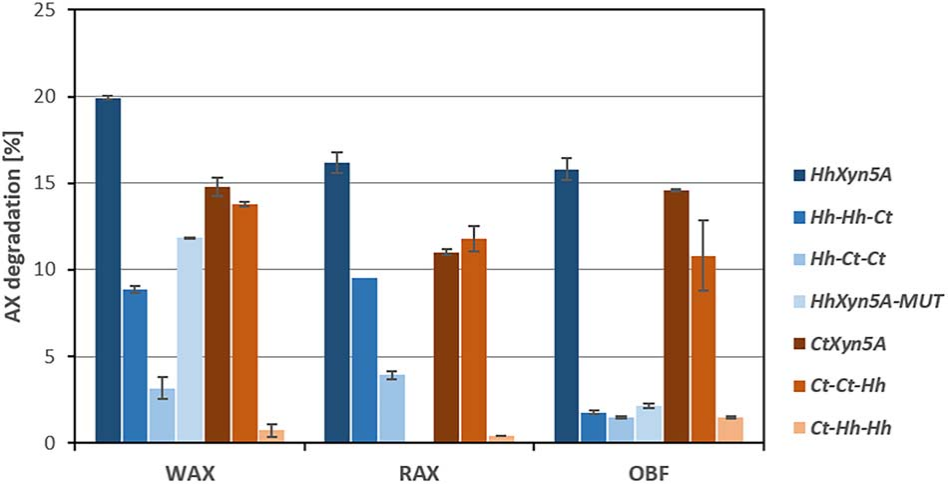
Enzymatic activity as percentage arabinoxylan (AX) degradation of total AX after 24 h on different substrates. WAX = wheat arabinoxylan; RAX = rye arabinoxylan; OBF = alkali soluble oat bran fibre. Reactions were performed in duplicates. *Hh*Xyn5A-MUT variant activity on RAX was not determined.

Hydrolytic activity of the enzyme variants on AX substrates was determined as reducing end formation with the di-nitrosalicylic acid (DNS) assay, measured after 24 h of incubation at 50 °C (Fig. 2). The reducing end formation was applied to calculate percent of AX degradation of total AX content in the respective substrate that all had an A/X ratio of 0.6. *Hh*Xyn5A and *Ct*Xyn5A demonstrated comparable activity levels on all substrates (Fig. 2) as previously observed (Norlander et al. 2023). Compared to the two-domain *Hh*Xyn5A variant (Fig. 1C) (Norlander et al. 2023), the domain-swapped *Hh-Ct-Ct* variant, with both linker and CBM6 swapped, displayed significantly lower activity on all substrates tested: wheat arabinoxylan (WAX), rye arabinoxylan (RAX), and alkali extracted oat bran fibre (OBF). Specifically, the *Hh-Ct-Ct* activity was only in the range of 9%–24% compared to the activity level of *Hh*Xyn5A, depending on the substrate used (Fig. 2). The effect on activity was less pronounced for the variant *Hh-Hh-Ct*, with only CBM6 swapped, but still demonstrated a substrate specific dependence with low activity on OBF. The activity level of the *Ct-Hh-Hh* variant, again with both linker and CBM6 swapped, was reduced to as little as 4%–10% of *Ct*Xyn5A (Fig. 2), depending on the type of substrate tested. On the other hand, the *Ct-Ct-Hh* variant*,* with only the CBM6 swapped*,* demonstrated comparable activity levels to the original *Ct*Xyn5A, on all three substrates (Fig. 2). This indicates that the influence of the CBM6-exchange was lower for the variants with the *Ct*GH5_34 catalytic module, while the variants including the *Hh*GH5_34 catalytic module demonstrated a notable reduction in activity by the domain exchange on all substrates. Additionally, the *Hh*Xyn5A-MUT variant, with four amino acid residues in cleft A of the *Hh*CBM6 mutated to the residues at the corresponding positions in *Ct*CBM6, demonstrated a similar activity level as the *Hh-Hh-Ct* variant on WAX and OBF substrates, indicating that that specific interactions were more important than the overall position of the CBM6.

**Fig. 3:**
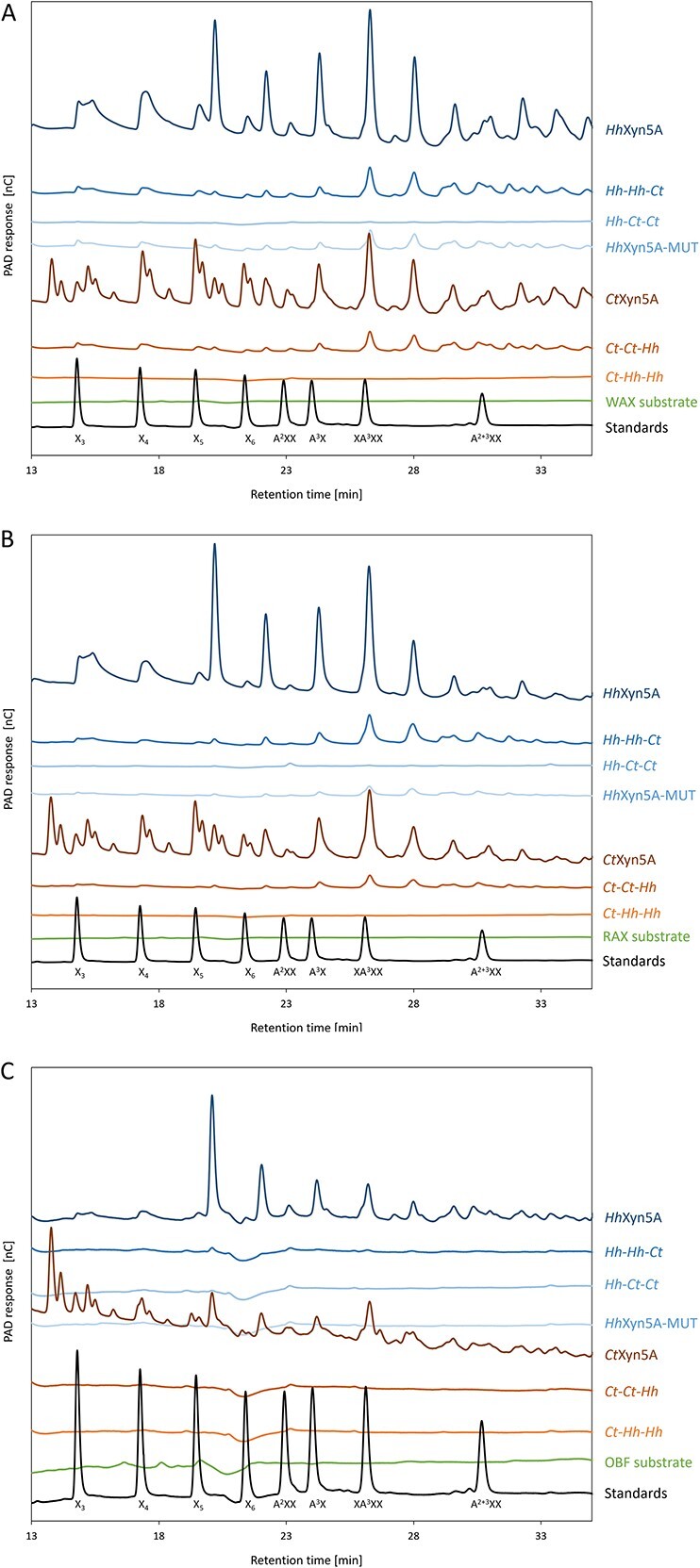
HPAEC-PAD chromatograms of oligosaccharide product profiles from enzyme *Hh*Xyn5A and *Ct*Xyn5A variants after 24 h using substrates (A) wheat arabinoxylan, (B) rye arabinoxylan, and (C) alkali extracted oat fibre. The chromatograms were zoomed in and the first few minutes of the elution profiles were omitted, in order to visualize hydrolysis products of interest. Arabinoxylo-oligosaccharide standards are represented in the black curve of the chromatogram. A = arabinose; X = xylose.

The oligosaccharide products of the enzymatic hydrolysis were analysed applying high performance anion exchange chromatography with pulsed amperometric detector (HPAEC–PAD) (Fig. 3). Both *Hh*Xyn5A and *Ct*Xyn5A produced broad product spectra from all substrates, generating peaks corresponding to elution times for various AXOS standards. It was also noted that *Ct*Xyn5A produced many additional products, seen as multiple unidentified peaks in the chromatograms, compared to the product profile from *Hh*Xyn5A. The *Ct-Ct-Hh* variant produced product spectra similar to *Hh*Xyn5A from WAX (Fig. 3A) and RAX (Fig. 3B) substrates, although at lower concentrations. Similar chromatogram peak patterns to *Hh*Xyn5A were also observed for the variants *Hh*-*Hh-Ct* and *Hh*-*Ct-Ct* on WAX and RAX, however at significantly lower concentrations (Supplementary Fig. S3), indicating that the complete set of interactions in *Ct*Xyn5A are necessary for the more complex product profile. No oligosaccharide products from any of the three substrates used could be detected applying HPAEC–PAD for the *Ct-Hh-Hh* variant. The HPAEC–PAD oligosaccharide product profile comparison supports the assumption that loss of substrate hydrolysis efficiency was a consequence of exchange of both the CBM6 and linker region for the two enzymes, especially for *Hh*Xyn5A variants including the *Hh*GH5_34 catalytic module. Additionally, it should be noted that *Hh*Xyn5A-MUT variant oligosaccharide product pattern was similar to the product profile from the original two-domain *Hh*Xyn5A enzyme (Supplementary Fig. S4), and did not change as a consequence of the introduced mutations (while the activity level was more similar to the *Hh-Hh-Ct* variant).

### Enzyme variant binding affinity to WAX

**Fig. 4:**
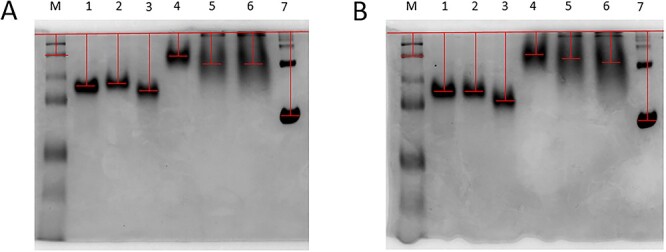
Affinity gel electrophoresis determination of enzyme variant binding affinity to wheat arabinoxylan (WAX). (A) Native PAGE with incorporated WAX, (B) native PAGE (control gel) without substrate. (M) Precision plus protein all blue Prestained protein standards (bio-rad) molecular-mass marker, (1) *Hh*Xyn5A, (2) *Hh-Hh-Ct*, (3) *Hh-Ct-Ct*, (4) *Ct*Xyn5A, (5) *Ct-Ct-Hh*, (6) *Ct-Hh-Hh*, (7) bovine serum albumin (BSA) fraction reference. The red lines indicate migration distance.

In order to investigate the CBM6 as well as the effect of swapping both linker and CBM6, on enzyme-substrate affinity, affinity gel electrophoresis was performed using WAX as substrate. The migration distances measured for all *Hh*Xyn5A and *Ct*Xyn5A enzyme variants (Fig. 4 and Table 2) did not differ substantially between the two native PAGE gels, with incorporated WAX and control gel without substrate, indicating very low binding affinity to the substrate for all enzyme variants. The calculated and normalized migration distance ratio (MDR) demonstrated a slight migration retardation and increased binding to WAX for the domain-swapped variants *Hh-Hh-Ct* (0.90) and *Hh-Ct-Ct* (0.88)*,* compared to original *Hh*Xyn5A (0.94), reflected as lower MDR values. Thus, the *Ct*CBM6 appears to display a somewhat increased binding to the substrate. In contrast, *Ct-Ct-Hh* and *Ct-Hh-Hh* variants displayed higher MDR values (1.24 and 1.09, respectively) compared to *Ct*Xyn5A (1.05), indicating slightly weaker binding of *Ct*Xyn5A domain-swapped variants to WAX.

**Table 2:** Affinity gel electrophoresis enzyme variant migration distances and resulting MDR values.

**protein**	**WAX**	**control**	**MDR**	**normalised MDR**	
	*migration distance, cm*			*based on BSA*	*based on protein standard*
second protein standard	0.44	0.50	0.88	0.93	1.00
*Hh*Xyn5A	1.12	1.25	0.90	0.94	1.02
*Hh-Hh-Ct*	1.08	1.27	0.85	0.90	0.97
*Hh-Ct-Ct*	1.24	1.48	0.84	0.88	0.95
*Ct*Xyn5A	0.50	0.50	1.00	1.05	1.14
*Ct-Ct-Hh*	0.67	0.57	1.18	1.24	1.34
*Ct-Hh-Hh*	0.68	0.66	1.03	1.09	1.17
BSA	1.83	1.93	0.95	1.00	1.08

WAX = wheat arabinoxylan; MDR = migration distance ratio; BSA = bovine serum albumin fraction.

### Amino acid interactions in the linker region

**Fig. 5:**
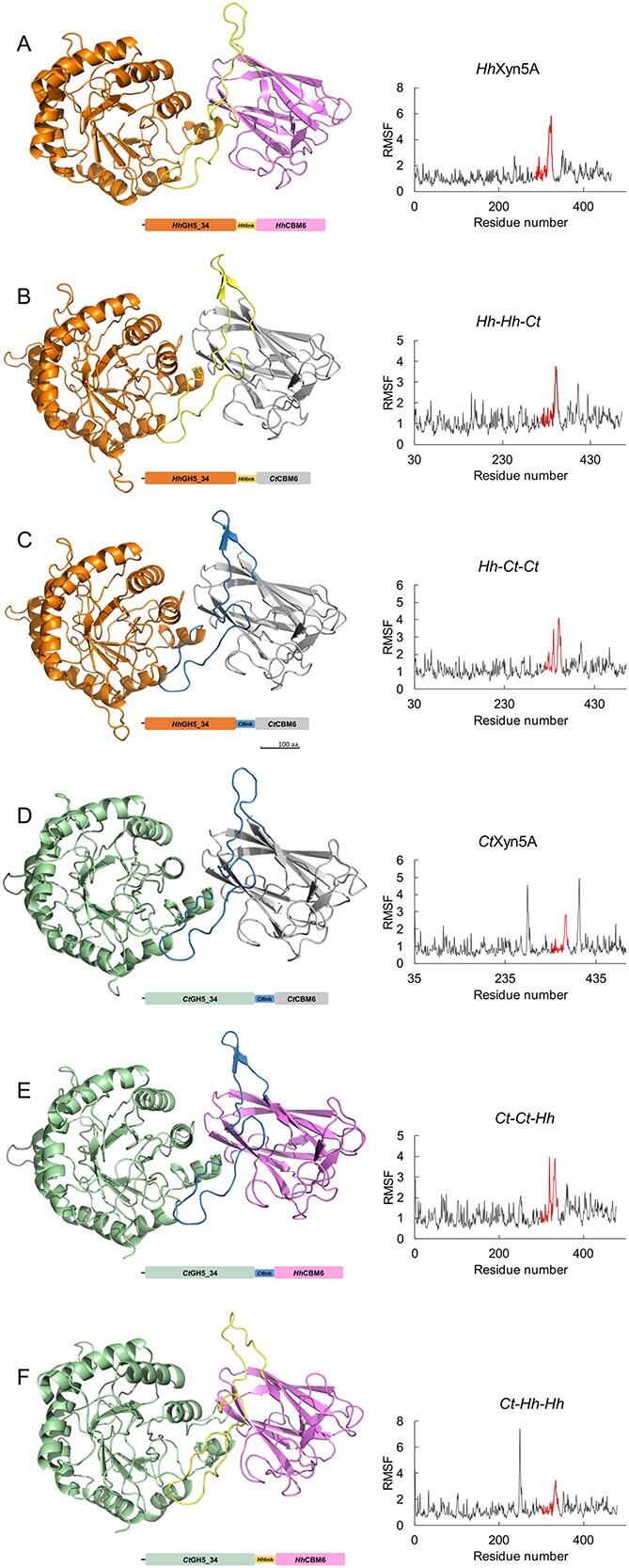
Enzyme variants structure homology models accompanied by schematic representation of the domain organization as well as the corresponding RMSF plots after a 50 ns molecular dynamic simulations. A) *Hh*Xyn5A. B) *Hh-Hh-Ct*. C) *Hh-Ct-Ct*. D) *Ct*Xyn5A. E) *Ct-Ct-Hh*. F) *Ct-Hh-Hh*. Graphical representations of structures were generated using PyMOL 2.5. The linker regions in RSMF plots are indicated in red.

To further investigate the potential interactions between the linker region, the catalytic GH5_34 domain and the CBM6, the domain-swapped variants were modelled and compared with *Ct*Xyn5A and *Hh*Xyn5A. All homology models were created using the three available structures of *Ct*Xyn5A (PDB 5LA2; PDB 2Y8K; PDB 5G56) as templates. Created variant structure models displayed stability after a 50 ns molecular dynamics (MD) simulation, with no major conformational changes observed (Fig. 5, RMSD plots shown in Supplementary Fig. S5). Potential polar interactions and π-interactions were identified between the linker region and the CBM6, as well as the catalytic domain for each enzyme variant structure (Supplementary Table S1). Both *Hh*Xyn5A domain-swapped variants displayed a few more interactions between the linker region and the CBM6 compared to the original *Hh*Xyn5A enzyme and the *Hh-Ct-Ct* variant additionally displayed more interactions between the linker region and the catalytic domain. The total number of differing amino acid interactions between the swapped variants and *Hh*Xyn5A was however similar for both *Hh-Hh-Ct* and *Hh-Ct-Ct*, either due to loss of interaction or formation of a new interactions, although *Hh-Ct-Ct* variant exhibited more differences. The root-mean-square fluctuation (RMSF) plot (Fig. 5) indicates significant fluctuations at the C-terminal region of the linker, peaking at 5.76 Å for *Hh*Xyn5A, 4.1 Å for *Hh-Ct-Ct*, and 3.74 Å for *Hh-Hh-Ct* variant. The reduced fluctuations may explain the increase in thermostability for *Ct*-variants. For the *Ct*Xyn5A variants, *Ct-Ct-Hh* displayed similar interactions compared to the original *Ct*Xyn5A enzyme, while *Ct-Hh-Hh* exhibited more differences, especially concerning interactions between the linker and the CBM6 domain. The RMSF plot of the *Ct*Xyn5A domain-swapped variants reveals that the highest fluctuation, reaching 7.42 Å, occurred at the C-terminal region of the linker in *Ct-Hh-Hh* variant. In contrast, *Ct-Ct-Hh* variant exhibited lower fluctuation (of 3.98 Å), which is lower than the fluctuations observed at the corresponding site in the *Ct*Xyn5A enzyme (4.95 Å fluctuation value) (Fig. 5).

## Discussion

To investigate the influence of CBM6 on hydrolytic activity and specificity of GH5_34 enzymes, a comparison of *Hh*Xyn5A and *Ct*Xyn5A with domain-swapped variants was performed (Table 3). The enzyme domain-swapped variant set was formed after interpreting the identified linker region as a structural element integrated with either the GH5_34 catalytic domain or the CBM6 domain. This assumption was based on the *Ct*Xyn5A structure analysis, which was indicating a stable conformation as well as a domain interface interaction network with the linker region (Correia et al. 2011). Production of the *Ct-Ct-Hh* and *Ct-Hh-Hh* variants resulted in comparatively low yields and domain splitting, and was negatively influenced by the exchange of the *Ct*CBM6 to *Hh*CBM6. Additionally, the aggregation observed for the *Hh-Ct-Ct* variant indicates that improper folding may occur as a result of swapping the linker region as well as the CBM6. This indirectly supports the assumption that the linker region of *Hh*Xyn5A and *Ct*Xyn5A is an important element for proper domain interaction and folding, of importance for storage stability in solution. Influence of the linker region on thermostability in the domain-swapped enzyme variants was, however, not observed. Exchange of the CBM6 domains, however led to improved thermostability in the variants including the more thermostable domain. A previous study on *Ct*Xyn5A suggests that the polar and apolar interactions of the linker region, make the catalytic domain and the CBM6 co-dependent (Correia et al. 2011). In the present study, the identified number of amino acid interactions in modelled *Hh*Xyn5A and *Ct*Xyn5A variant structures did not differ much between the enzymes, but as the linker region is conserved, the interactions in conserved parts can be restored even after a linker exchange. Thus, the conclusion that the linker substantially influences the position of the domains, activity and thermostability of the GH5_34 enzymes is valid also in this study. *Ct-Ct-Hh* displayed the least differences in interactions, when comparing the original enzyme with the domain-swapped variants, and retained most of the activity. In contrast, *Ct-Hh-Hh* variant that displayed most differences in linker interactions, demonstrated both protease susceptibility and loss of enzymatic activity. The comparison of RMSF plots indicates that the linker region in *Hh-Hh-Ct* and *Hh-Ct-Ct* variants is more stable than in the original *Hh*Xyn5A, while the swap of linker region and CBM6 connected to the *Ct*Xyn5A catalytic domain led to significantly higher fluctuation in the linker region of the *Ct-Hh-Hh* variant, that also displayed the lowest activity.

The enzyme variants *Hh*-*Hh-Ct* and *Ct-Ct-Hh* with the linker region connected to the natively corresponding catalytic domain displayed higher activity on AX substrates, than *Hh*-*Ct-Ct* and *Ct-Hh-Hh* variants where the CBM6 and linker region originated from the same enzyme but were not of the same origin as the catalytic module. These constructs demonstrated only limited activity, and when applying HPAEC–PAD no oligosaccharides could be detected. The *Ct-Ct-Hh* variant displayed high activity, comparable to the original *Ct*Xyn5A, however interestingly displayed product spectra which instead were more similar to the product pattern of *Hh*Xyn5A. This indicates that the CBM6 in *Ct*Xyn5A is at least partially responsible for activity and specificity of the enzyme allowing formation of additional shorter products. This supports the hypothesis in our previous study (Norlander et al. 2023), that the CBM6 is affecting the differences in product pattern. The linker region may, on the other hand, be responsible for the localisation of the binding site of CBM6 in relation to the catalytic GH5_34 domain, which may affect the ability of the CBM6 to act in a productive manner together with the catalytic domain.

The *Hh*Xyn5A-MUT variant demonstrated a similar activity level as the *Hh*-*Hh*-*Ct* variant and was notably less active than both the *Hh*Xyn5A and *Ct*Xyn5A enzymes, while the *Hh*Xyn5A-MUT product pattern was similar to *Hh*Xyn5A, indicating release of fewer types of oligosaccharides, and the mutations (introduced based on residues in *Ct*CBM6) did hence not improve the number of products released.

The observed limited activity of *Hh*-*Ct*-*Ct* and *Ct*-*Hh*-*Hh*, with no oligosaccharide products detected by applying HPAEC–PAD although showing activity applying the DNS assay, indirectly indicates release of longer oligosaccharides than the maximum size detectable by the chromatographic method. Hydrolysis products, released by GH5_34 arabinoxylanase, and longer than detectable by HPAEC-PAD has been reported and identified applying MS (Rudjito et al. 2023). Overall, the enzyme variants activity results demonstrate the importance of the linker region in hydrolysis, where the inter-domain linker region can play a significant role in positioning the CBM6 in relation to the catalytic site of the catalytic domain, thereby determining the efficiency in oligosaccharide production.

*Ct*CBM6 has previously shown low to no binding affinity to longer xylan substrates, however the domain has been found to be necessary for binding shorter oligosaccharides to the enzyme, specifically by the aromatic residues Trp424 and Phe478 (Correia et al. 2011). In the present study, the CBM6 domains in both *Hh*Xyn5A and *Ct*Xyn5A enzyme variants showed low binding affinity to WAX as indicated by the minor differences in enzyme migration measured between the affinity electrophoresis gel with incorporated substrate and the control gel. A slight increase in retardation could be observed for *Hh-Hh-Ct* and *Hh-Ct-Ct* variants, as well as a decrease in retardation for *Ct–Ct-Hh* and *Ct-Hh-Hh* variants, indicating that the *Hh*CBM6 has an even lower affinity to WAX compared to *Ct*CBM6. In our previous study (Norlander et al. 2023), characterisation of *Hh*Xyn5A showed that *Hh*CBM6 lacks the important aromatic residues that forms the classical binding cleft for XOS substrates, suggesting that *Hh*Xyn5A has lower affinity to XOS compared to *Ct*Xyn5A and is possibly less likely to hydrolyse XOS substrates. This assumption is further supported by the lack of the shorter oligosaccharide products from *Hh*Xyn5A, which are detected in the *Ct*Xyn5A enzyme HPAEC–PAD spectra. The *Hh*Xyn5A-MUT variant with mutated *Hh*CBM6, is re-creating the typical CBM6 aromatic cleft A of *Ct*Xyn5A and the hydrogen bonding that is missing in native *Hh*CBM6, but still demonstrated a similar product pattern to *Hh*Xyn5A, which indicates that the aromatic stacking interactions in cleft A are not the sole factor determining protein-ligand interaction and product profile. The analysis and overall results suggest that *Hh*CBM6 may have an effective stabilising function of the catalytic domain or an additional function in a potential cellulosome complex, functioning together with other domains to break down recalcitrant plant material. Although it was previously shown that the *Hh*CBM6 is essential for hydrolytic activity, as the single catalytic domain is inactive (Norlander et al. 2023), it could be suggested that the inter domain linker region may be as important as the CBM6 for substrate association to the catalytic domain in the *Hh*Xyn5A arabinoxylanase.

## Material and methods

### Construction and cloning of *Hh*Xyn5A and *Ct*Xyn5A enzyme variants

Enzyme variants were constructed by combining the catalytic GH5_34 domain with the linker region and CBM6 analogously to the domain organisation of the truncated two-domain *Hh*Xyn5A and *Ct*Xyn5A variants (Fig. 1C). Sequences of combined enzymes modules originated from GH5_34 arabinoxylanases *Hh*Xyn5A-FULL (GenBank NLC19267.1 (Campanaro et al. 2020)) and *Ct*Xyn5A-FULL (RefSeq WP_003513669.1 (Wilson et al. 2013)). Initial characterisation of domain organisation (Norlander et al. 2023) through NCBI Conserved Domain Database 3.17 (Lu et al. 2020) was further investigated re-evaluating enzyme sequence module boundaries by comparison of the *Hh*Xyn5A tertiary structure model (Norlander et al. 2023) with the *Ct*Xyn5A structure (PDB 2Y8K). Protein sequences were aligned defining the amino acid conservation and visualized using Jalview 2.11 (Waterhouse et al. 2009). Sequences encoding signal peptides predicted with the SignalP 6.0 server (Teufel et al. 2022) were omitted. Four constructed domain-swapped enzyme variants *Hh*-*Hh*-*Ct, Hh-Ct-Ct, Ct-Ct-Hh* and *Ct-Hh-Hh* (Fig. 1C) were cloned in frame with an N-terminal His_6_-tag encoding sequence. Enzyme mutated variant *Hh*Xyn5A-MUT was constructed by mutating four amino acid residues (H376W, D430F, G431Q, K459N) in the CBM6 sequence to the residues at the corresponding positions in CBM6 of the *Ct*Xyn5A variant (Fig. 1C) and cloned in frame with an N-terminal His_6_-tag encoding sequence. All constructed variant sequences were vector-adapted maintaining the native codon landscape, synthesised and cloned (Bio-Cat, Germany) in pET-21b(+) vectors (Merck).

### Production and purification of recombinant enzyme variants

Heterologous overexpression in *E. coli* inducing with isopropyl β-D-galactopyranoside and purification applying IMAC of all enzyme variants was performed as previously described (Norlander et al. 2023) for *Hh*Xyn5A and *Ct*Xyn5A variants. The purified proteins were dialyzed at 4 °C through 3,500 Da MWCO regenerated cellulose membranes against formulation buffer (50 mM HEPES-HCl pH 6.5 (RT)) at 1:5000 volume ratio. If domain splitting of recombinant proteins was observed purification was performed using SIGMA*FAST* Protease Inhibitor Tablets (Merck) for protease activity inhibition. Buffer exchange to formulation buffer, while maintaining protease inhibition was performed applying group separation with HiTrap Desalting 5 mL (16 × 25 mm) columns (Cytiva) loading 10% (v/v) injection volume onto two tandem connected desalting columns at 0.5 mL/min loading flowrate. The commercial *Ct*Xyn5A (product number CZ00601) produced in *E. coli* and purified applying IMAC was purchased from NZYTech. Protein samples were filtered through 0.22 *μ*m pore size regenerated cellulose filters. The protein concentration was determined by measuring absorbance at 280 nm (assuming A_280_ 1 = 1 mg/mL as well as considering absorption coefficients (Supplementary Table S2) predicted with the ProtParam tool (Gasteiger et al. 2005)) using BioSpec-nano spectrophotometer (Shimadzu). Purity and integrity of proteins were analyzed by visualization applying 4%–15% glycine–SDS–PAGE (Bio-Rad). If domain splitting of recombinant proteins was observed, purified fragments were excised from stained SDS–PAGE gel and analysed applying MS (Bruker Daltonics).

### Enzyme stability

Enzyme variant *T*_m_ was estimated in reaction buffer applying nanoDSF using a Prometheus NT.48 instrument (NanoTemper Technologies). Standard grade glass capillaries (NanoTemper Technologies) were filled with enzyme solution at a concentration of 0.1–0.2 mg/mL. Thermal unfolding was performed with a temperature gradient between 20 and 95 °C at a 1 °C/min ramp rate and adjusting excitation power to 40%. *T*_m_ was determined from the first derivative of the absorbance ratio 350/330 nm and was identified automatically by the instrument software PR.ThermControl 2.0 (NanoTemper Technologies).

### Enzyme activity assay

Enzyme activity was measured by monitoring reducing end formation from different substrates with DNS assay (Miller 1959). Substrates were suspended at 10 mg/mL in reaction buffer (10 mM HEPES-HCl pH 6.5 (RT)) and added to 1.5 mL tubes at 9:1 volume ratio of substrate and enzyme preparation of 0.2 mg/mL. The reactions were incubated in thermoshakers at 50 °C with shacking at 500 rpm for 24 h (Norlander et al. 2023). Samples were collected to be used for DNS assay and oligosaccharide analysis. DNS reagent was added to samples at a 1:1 volume ratio to stop the reaction and the samples were then boiled for 10 min and chilled on ice, before measuring absorbance at 540 nm with Multiskan GO microplate spectrophotometer (Thermo Fisher Scientific). The reducing end formation was calculated based on a D-xylose standard curve and the results were divided with the theoretical reducing end formation of total AX degradation for each substrate, resulting in a hydrolysis percentage of total AX. Samples collected for oligosaccharide analysis were boiled for 10 min, subsequently diluted and filtered through 0.2 *μ*m pore size PTFE filters.

Substrates including soluble wheat arabinoxylan (WAX) (low viscosity) and soluble rye arabinoxylan (RAX) were purchased from Megazyme. Alkali soluble oat bran fibres (OBF) were extracted (Schmitz et al. 2022) from an insoluble oat fibre bran fraction obtained from oat processing, provided by Lantmännen Oats (Sweden) in 2020 from their production site oat mill in Kimstad (Sweden). AX content of the extracted oat fibres was determined through acid hydrolysis and quantification of arabinose and xylose through HPAEC–PAD as previously described (Schmitz et al. 2022). AX content of WAX, RAX and OBF were 95, 90, and 5.2%, respectively. All AXOS substrates had a similar A/X ratio of 0.6.

### Analysis of AX hydrolysis oligosaccharide product profile

The oligosaccharide product profiles after the enzymatic reactions were investigated applying HPAEC—PAD with a Dionex ICS-6000 system (Thermo Fischer Scientific) using a Dionex CarboPac PA300 (250 × 2 mm, 4 μm) analytical and a guard 50 × 2 mm, 4 μm) columns (Thermo Fisher Scientific). The separation was performed using a constant mobile phase composition of 100 mM NaOH at 0.25 mL/min, and a linear gradient from 0 to 30 min of 0–120 mM sodium acetate and thereafter a constant concentration of 160 mM sodium acetate until 45 min. AXOS standards xylobiose (X^2^), xylotriose (X^3^), xylotetraose (X^4^), xylopentaose (X^5^), xylohexaose (X^6^), 3^2^-α-L-arabinofuranosylxylobiose (A^3^X), 2^3^-α-L-arabinofuranosyl-xylotriose (A^2^XX), 3^3^-α-L-arabinofuranosyl-xylotetraose (XA^3^XX), 2^3^-α-L-arabinofuranosyl-xylotetraose (XA^2^XX), and 2^3^,3^3^-di-α-L-arabinofuranosyl-xylotriose (A^2 + 3^XX) were purchased from Megazyme.

### Affinity gel electrophoresis

Affinity gel electrophoresis was performed (Cockburn et al. 2023) in order to investigate the domain swapping effect on enzyme-substrate affinity comparing with determined *Hh*Xyn5A and *Ct*Xyn5A variant binding affinity to WAX. Native 10% PAGE gels were prepared incorporating 0,1% (w/v) WAX or without substrate. Running buffer (300 mM Tris–HCl, pH 8.8 (RT)) was used for protein separation performing PAGE at constant 10 V/cm. Native PAGE gels were stained with Coomassie Brilliant Blue R-250 Staining Solution (Bio-Rad) and detained using distilled water. Bovine serum albumin fraction (BSA) (Sigma–Aldrich) was used as non-binding reference protein. GelDoc Go imaging system (Bio-Rad) was used for gel documentation. The migration distance for the protein in native PAGE with incorporated WAX was divided with the migration distance in native PAGE control gel without WAX substrate resulting in calculated MDR.

### Homology modelling for evaluation of the linker region amino acid interactions

Homology models of domain-swapped enzyme variants were generated using YASARA 21.12.19 program (YASARA Biosciences) in order evaluate the linker region interactions with the catalytic GH5_34 domain and CBM6 by comparing modelled structures of enzyme variants. The models were created by entering the respective amino acid sequence for each enzyme variant and running the macro “hm_build”, a script which searches PDB for suitable templates and creates a model based on the four best aligned templates. The resulting templates suggested by the script and used for all models were the three available structures of *Ct*Xyn5A (PDB 5LA2; PDB 2Y8K; PDB 5G56). All models were subjected to molecular dynamics simulation for 50 ns at 298 K and pH 6.5 using YASARA program. The cubic simulation cell was chosen to be 20 Å larger than the protein in all directions, accommodating explicit water molecules as the solvent and Na^+^ and Cl^−^ as counter ions. Snapshots of the simulation were stored at regular intervals of 100 ps, to investigate the systems dynamics over time. Energy calculations were performed using the AMBER14 force field, providing insights into the distribution of energy and interactions within the simulated system. RMSD and RMSF plots were generated for each simulation. The energetically minimized snapshots of the structures from the simulation were superimposed and structurally compared evaluating hydrogen bonding and π-interactions as well as generating graphical representation of structures using PyMOL 2.5 (Schrödinger). Amino acid numbering correspond to the respective enzyme tertiary structure numbering.

## Supplementary Material

Supplementary_cwae048

## Data Availability

The data underlying this article are available in the article and in its online supplementary material.
