# Supplementary material for: Inter domain linker region affects properties of CBM6 in GH5_34 arabinoxylanases and alters oligosaccharide product profile
Source: Glycobiology. 2024 Jul 10;34(8):cwae048. doi: 10.1093/glycob/cwae048 (PMC11246198; doi:10.1093/glycob/cwae048)
Supplement: Supplementary_cwae048 [file supplementary_cwae048.docx]

Supplementary material


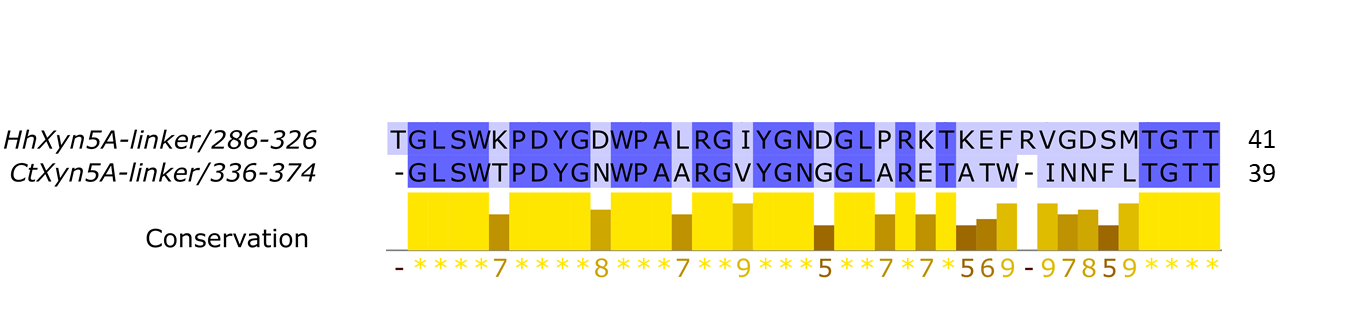


**Figure** **S1**. Sequence alignment of *Hh*Xyn5A and *Ct*Hyn5A enzymes linker region. The location (residue number) in the amino acid sequence in the full-length enzyme is indicated after the enzyme name. Dark blue columns represent identical residues, whereas light blue represents residues with a lower sequence similarity.


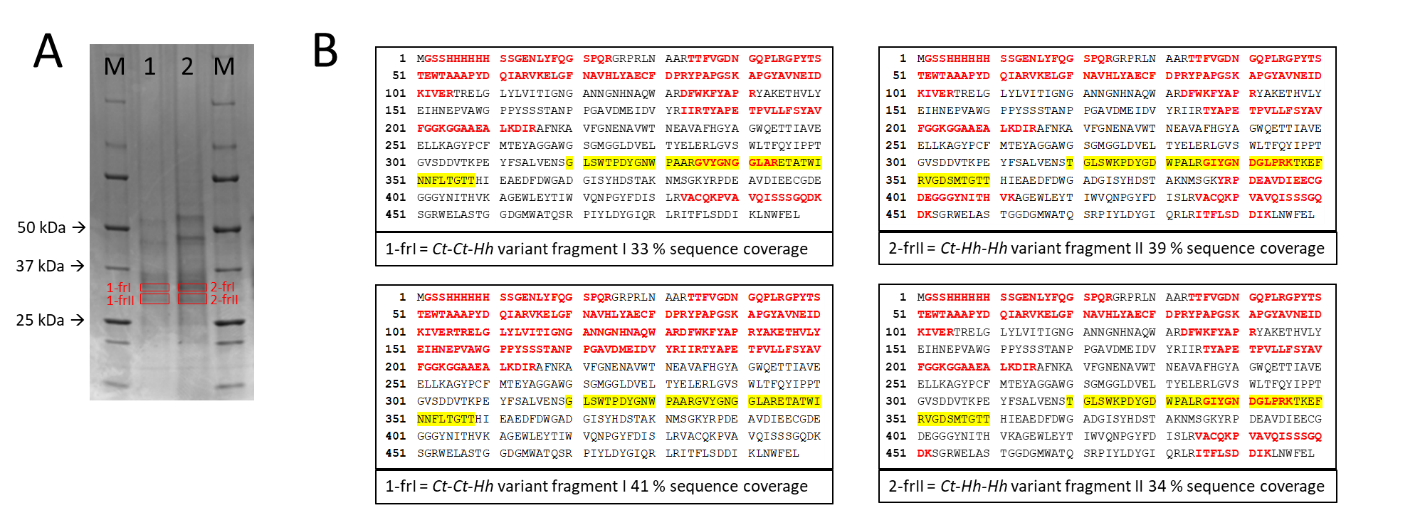


**Figure** **S2**. (**A**) SDS–PAGE of recombinant enzyme *Ct-Ct-Hh* and *Ct-Hh-Hh* variants after purification. (M) – Precision Plus Protein Unstained Standards (Bio-Rad) molecular-mass marker, (1) *Ct-Ct-Hh*, (2) *Ct-Hh-Hh*. Domain split variant fragment samples excised from SDS–PAGE gel is marked in red indicating sample name. (**B**) *Ct-Ct-Hh* and *Ct-Hh-Hh* variant fragment identification, applying mass spectrometry, results. Red text represents amino acid sequence covered by identified oligopeptides. The linker region sequence is highlighted in yellow.


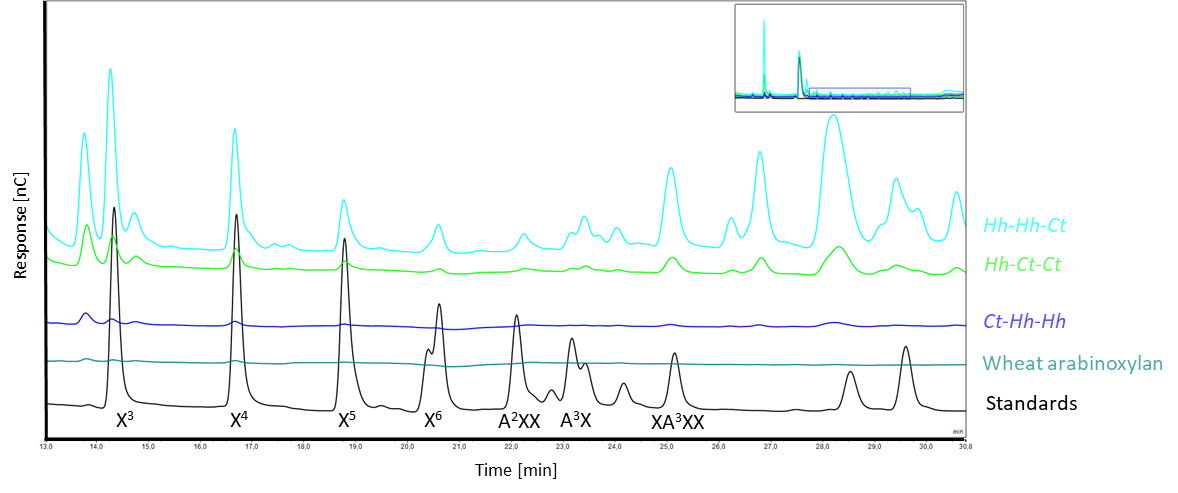


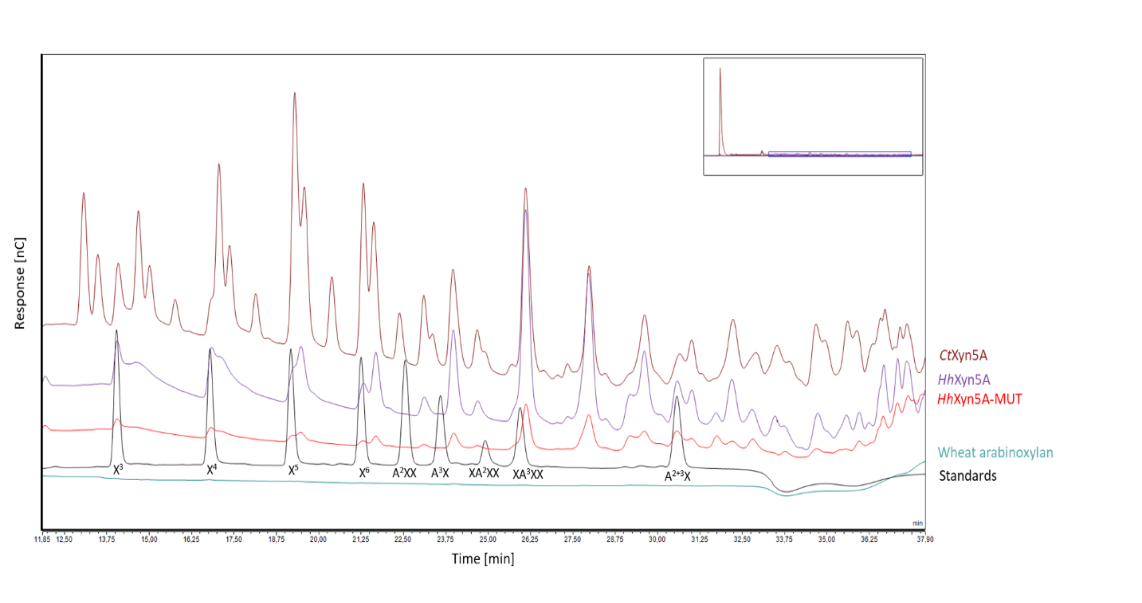
**Figure** **S3**. HPAEC–PAD chromatogram of oligosaccharide product profiles from the enzyme variants with CD, linker and CBM from *Hh*Xyn5A/*Ct*Xyn5A. The 24 h incubations using wheat arabinoxylan are shown. The chromatograms were zoomed in and the first few minutes of the elution profiles were omitted, in order to visualize hydrolysis products of interest. Arabinoxylo-oligosaccharide standards are represented in the black curve of the chromatogram. A = arabinose; X = xylose.

**Figure** **S4**. HPAEC–PAD chromatogram of oligosaccharide product profiles from the *Hh*Xyn5A and *Ct*Xyn5A enzyme variants after 24 h incubation with wheat arabinoxylan. The chromatograms were zoomed in and the first few minutes of the elution profiles were omitted, in order to visualize hydrolysis products of interest. Arabinoxylo-oligosaccharide standards are represented in the black curve of the chromatogram. A = arabinose; X = xylose.


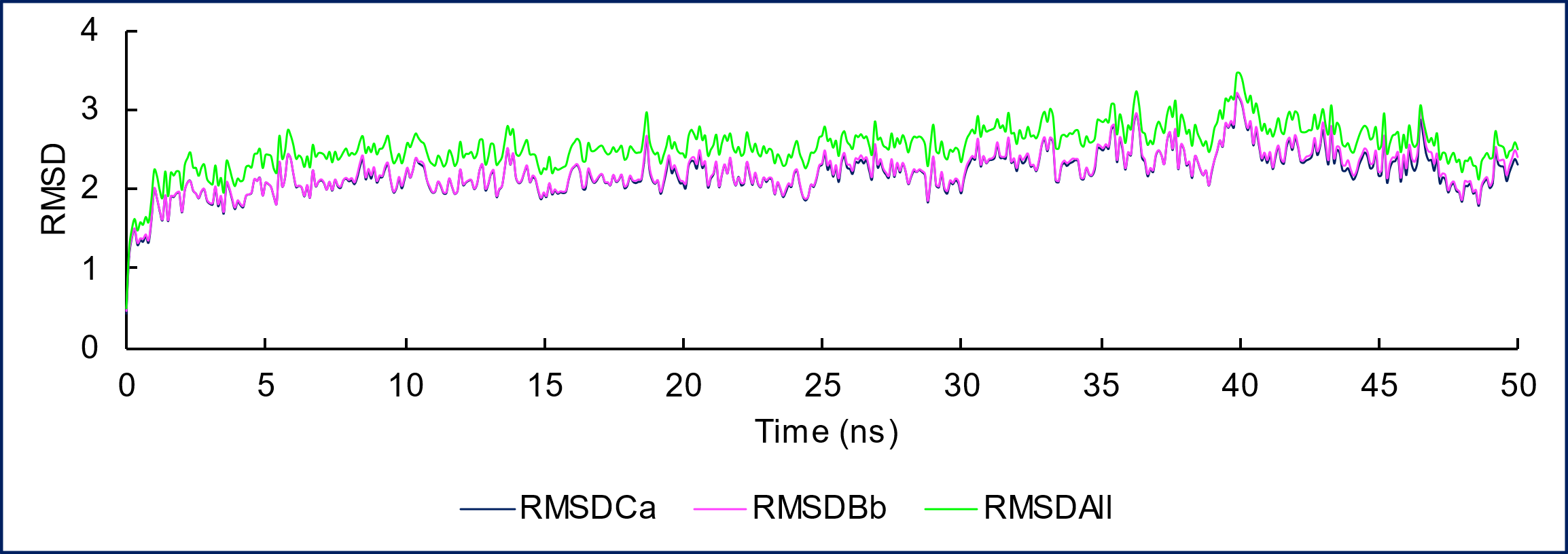


A

B


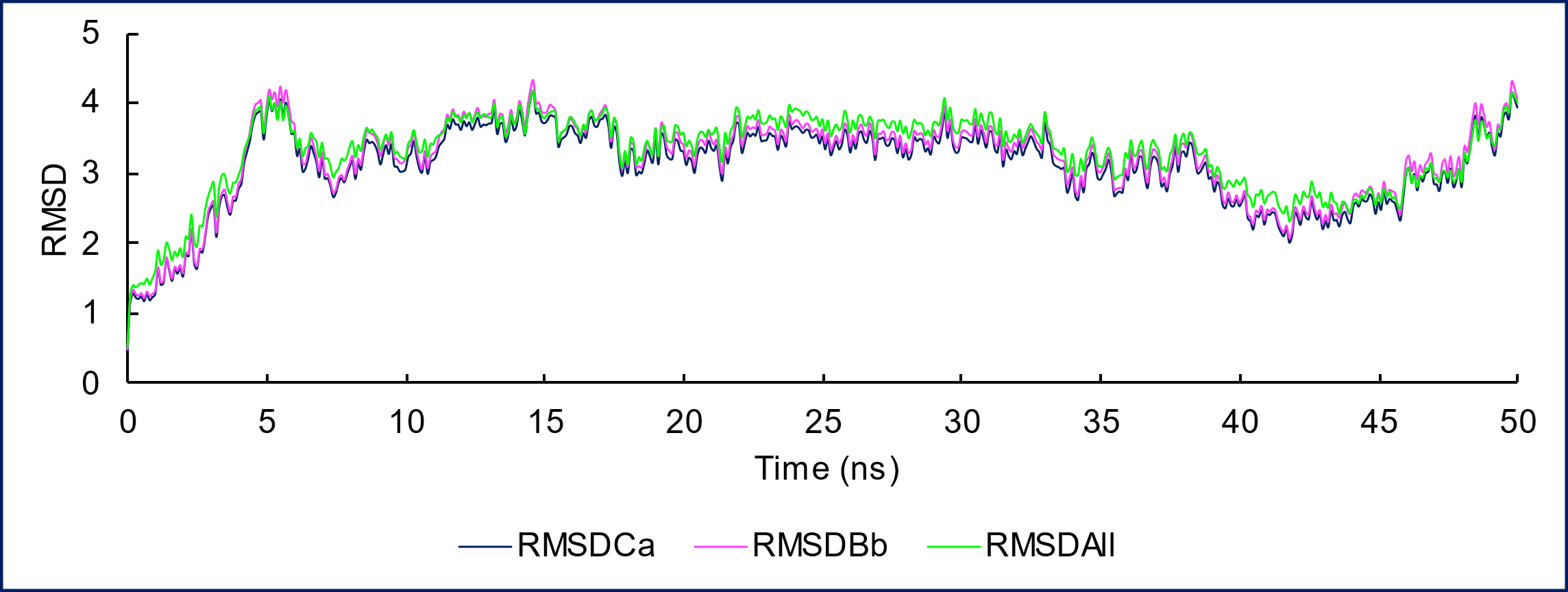


C


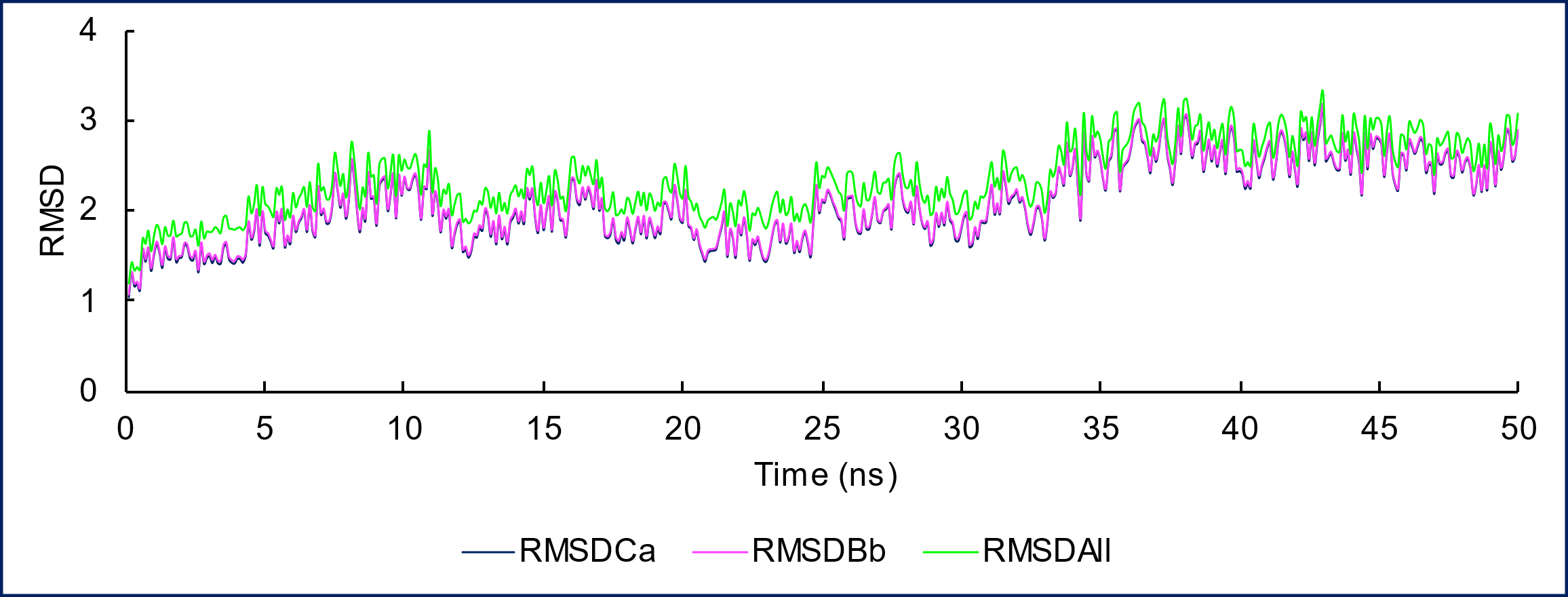


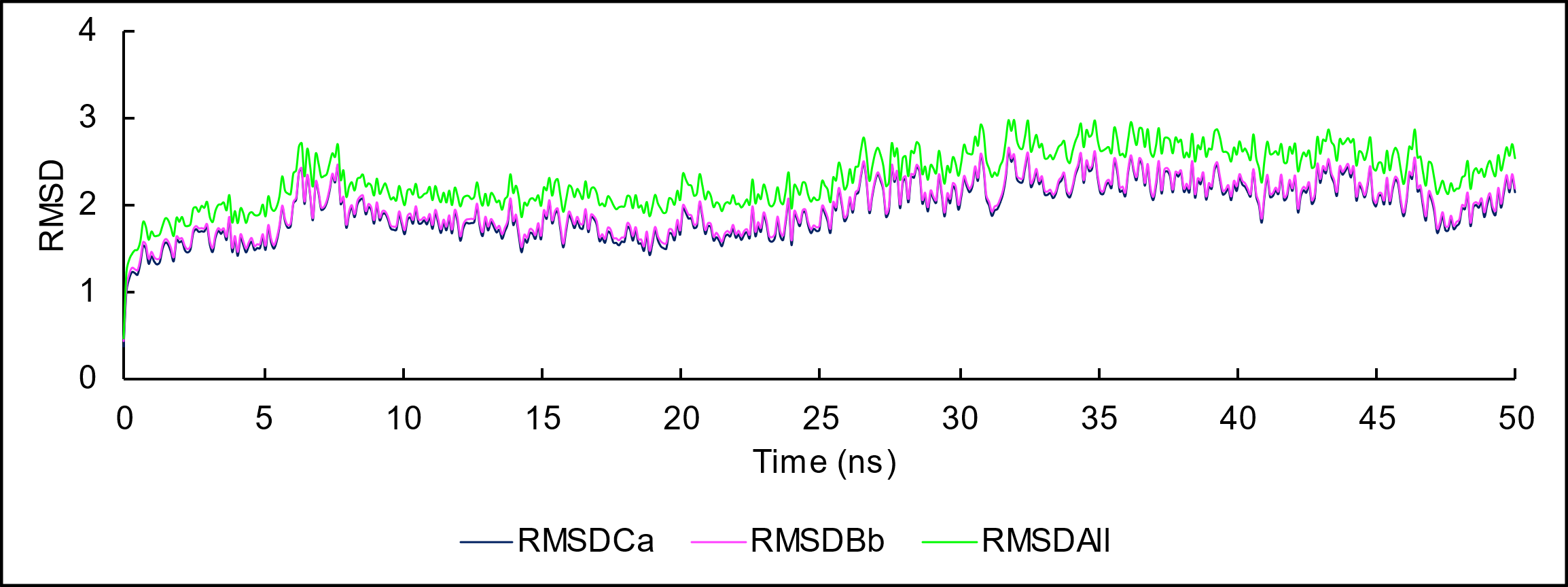


D

**Figure** **S5**. RMSD plots after a 50 ns molecular dynamics simulation of the *Hh*Xyn5A and *Ct*Xyn5A enzyme variants. (**A**) *Hh-Hh-Ct*. (**B**) *Hh-Ct-Ct*. (**C**) *Ct-Ct-Hh*. (**D**) *Ct-Hh-Hh*. Each figure displays the solute RMSD from the starting structure. The plot shows RMSD of Calpha = RMSDCa in black; backbone = RMSDBb in pink, and all-heavy atom = RMSDAll in green.

**Table SI**. Enzyme variant inter domain linker region amino acid residue interactions with catalytic GH5_34 domain and CBM6. The amino acid residue interactions with the catalytic domain are highlighted in bold with dark green background and interactions with CBM6 are highlighted in italics with light green background. The residue numbering of the linker region in the respective enzyme is indicated after the variant name. Amino acid interactions shown on the same row in the table are located at the corresponding position when comparing the superimposed tertiary structure model of the respective enzyme variant (only between the same catalytic domain). Hydrogen bonding and π-interactions were identified in the structure homology models using PyMOL 2.5.

| ***Hh*Xyn5A** (286–326) | ***Hh-Hh-Ct*** (319–359) | ***Hh-Ct-Ct*** (319–357) | ***Ct*Xyn5A** (336–374) | ***Ct-Ct-Hh*** (300–338) | ***Ct-Hh-Hh*** (300–340) |
| --- | --- | --- | --- | --- | --- |
| *Hydrogen bonding* | | | | | |
| **T286 ··· I282** | **T319 ··· I315** | **G319 ··· V316** | NB | NB | *T300 ··· E297, E398* |
| **G287 ··· V283, E284** | **G320 ··· V316, E317** | NB | **G336 ··· V332** | **G300 ··· V296** | *G301 ··· T400* |
| NB | **L321 ··· V316** | **L320 ··· V316** | **L337 ··· S335** | **L301 ··· S299** | **L302 ··· S299** |
| **W290 ··· G36** | NB | **W322 ··· L68** | **W339 ··· G85** | **W303 ··· G49** | **W304 ··· G49** |
| **D293 ··· N38, N7** | **D326 ··· N71, G69, N40, R24** | **D325 ··· R24, N40, G69, N71** | **D342 ··· R40, N56, N87** | **D306 ··· R4, N20, N51** | **D307 ··· R04, N20, N51** |
| **Y294 ··· G77** | **Y327 ··· G110** | **Y326 ··· R24, G110** | **Y343 ··· G126** | **Y307 ··· G90, R4** | **Y308 ··· R04** |
| **W297 ··· E284** | NB | NB | **W346 ··· E333** | NB | **W311 ··· E297** |
| *R301 ··· D334, I340, E384* | NB | *R333 ··· D365, V371, E415* | *R350 ··· D382, V388, E432* | *R214 ··· I351, E396* | **R315 ··· E297** |
| *G302 ··· W335* | *G335 ··· W368* | *G334 ··· W366* | *G351 ··· W383* | *G315 ··· W347* | *G316 ··· W349* |
| NB | *I336 ··· W368* | NB | **A359 ··· Y298** | **A323 ··· Y262** | NB |
| NB | NB | NB | *R360 ··· R436* | *R324 ··· D344,D346, T398, W400* | *R325 ··· T400, D346, D348* |
| NB | NB | **A342 ··· Y282** | *E361 ··· R436* | E325 ··· Q402 | NB |
| *R311 ··· T386, W388* | *R344 ··· T419, R421* | *R343 ··· R419, D363, T417* | *T362 ··· R436, R438* | NB | *T327 ··· W402* |
| NB | NB | *E344 ··· R421* | NB | NB | *K328 ··· L479* |
| *T313 ··· W388, Q390* | *T346 ··· Y428* | *T345 ··· Y426* | *W365 ··· P515* | *W329 ··· N403* | *F330 ··· N405* |
| NB | *K347 ··· P425, Y427, N424* | *A346 ··· N422* | *T371 ··· R438* | NB | NB |
| NB | *E348 ··· R423, N424* | NB | NB | *T337 ··· H339* | *T339 ··· H341* |
| NB | NB | *W348 ··· N422* | *T374 ··· R438, L513* | *T338 ··· L477* | *T340 ··· L479* |
| NB | *G357 ··· S499* | NB |  | | |
| NB | *T359 ··· L498* | *T357 ··· L496* |  |  |  |
| *π-interactions* | | | | | |
| **Y294 – K33** | **Y327 – K66** | **Y326 – K66** | NB | **W303 – W271** | NB |
| NB | NB | **W322 – W291** | **Y343 – K82** | **Y307 – K46** | **Y308 – K46** |

NB = no corresponding bond

**Table SII**. Predicted extinction coefficients for the enzyme variants.

| **enzyme variant** | **extinction coefficient (M^-1^ cm^-1^)** | ***ε*_0.1%_** |
| --- | --- | --- |
| *Hh*Xyn5A | 125710 | 2.272 |
| *Hh-Hh-Ct* | 125710 | 2.279 |
| *Hh-Ct-Ct* | 131210 | 2.395 |
| *Hh*Xyn5A-MUT | 131210 | 2.371 |
| *Ct*Xyn5A | 136710 | 2.481 |
| *Ct-Ct-Hh* | 136710 | 2.482 |
| *Ct-Hh-Hh* | 131210 | 2.367 |
